# Predictive nomograms based on gamma-glutamyl transpeptidase to prealbumin ratio for prognosis of hepatocellular carcinoma patients without microvascular invasion

**DOI:** 10.1186/s12885-024-12387-3

**Published:** 2024-05-21

**Authors:** Mingxiu Ma, Kailing Xie, Tianqiang Jin, Feng Xu

**Affiliations:** Department of General Surgery, Shengjing Hospital of China Medical University, Shenyang, Liaoning, China

**Keywords:** Gamma-glutamyl transpeptidase, Hepatocellular carcinoma, Prealbumin, Prognosis, Nomogram

## Abstract

**Background:**

Hepatocellular carcinoma (HCC) presents a significant threat to individuals and healthcare systems due to its high recurrence rate. Accurate prognostic models are essential for improving patient outcomes. Gamma-glutamyl transpeptidase (GGT) and prealbumin (PA) are biomarkers closely related to HCC. This study aimed to investigate the predictive value of the GGT to PA ratio (GPR) and to construct prognostic nomograms for HCC patients without microvascular invasion.

**Methods:**

We retrospectively analyzed data from 355 HCC patients who underwent radical hepatectomy at Shengjing Hospital of China Medical University between December 2012 and January 2021. Patients were randomly assigned to a training cohort (*n* = 267) and a validation cohort (*n* = 88). The linearity of GPR was assessed using restricted cubic spline (RCS) analysis, and the optimal cut-off value was determined by X-tile. Kaplan–Meier survival curves and log-rank tests were used to investigate the associations between GPR and both progression-free survival (PFS) and overall survival (OS). Cox multivariate regression analysis identified independent risk factors, enabling the construction of nomograms. Time-dependent receiver operating characteristic (ROC) and calibration curves were used to evaluate the accuracy of the nomograms. Decision curve analysis (DCA) assessed the predictive value of the models.

**Results:**

Patients were categorized into GPR-low and GPR-high groups based on a GPR value of 333.33. Significant differences in PFS and OS were observed between the two groups (both *P* < 0.001). Cox multivariate analysis identified GPR as an independent risk factor for both PFS (OR = 1.80, 95% CI: 1.24–2.60, *P* = 0.002) and OS (OR = 1.87, 95% CI: 1.07–3.26, *P* = 0.029). The nomograms demonstrated good predictive performance, with C-index values of 0.69 for PFS and 0.76 for OS. Time-dependent ROC curves and calibration curves revealed the accuracy of the models in both the training and validation cohorts, with DCA results indicating notable clinical value.

**Conclusions:**

GPR emerged as an independent risk factor for both OS and PFS in HCC patients without microvascular invasion. The nomograms based on GPR demonstrated relatively robust predictive efficiency for prognosis.

## Introduction

Hepatocellular carcinoma (HCC) is one of the most prevalent and life-threatening malignancies globally and ranks third in cancer-related mortality [1]. Currently, surgical resection remains the primary therapeutic modality for HCC treatment. However, despite achieving a 5-year survival rate of approximately 70%, the high recurrence rate, which can reach up to 70%, remains a significant challenge [2, 3]. Thus, there is a critical need for robust preoperative prognostic tools to guide clinical decision-making in HCC treatment.

In recent years, biomarkers such as albumin and gamma-glutamyl transpeptidase (GGT) have emerged as pivotal factors for predicting HCC prognosis [4, 5]. GGT, widely utilized as a biomarker for hepatobiliary diseases and liver cirrhosis, typically maintains a normal range of 0–60 U/L [6]. Elevated GGT levels often signify adverse clinicopathological features in HCC patients [7]. On the other hand, prealbumin (PA), synthesized in the liver, plays a crucial role in blood transport [8]. With a shorter half-life compared to albumin, PA provides a rapid reflection of the patient's nutritional status [9]. Low PA levels indicate malnutrition and compromised liver function. Several studies have suggested that diminished preoperative PA levels may serve as a predictor of unfavourable outcomes in HCC patients undergoing surgical resection [10–12].

The simultaneous presence of elevated GGT levels and reduced PA levels indicates compromised liver function and a greater likelihood of poor outcomes. Consequently, the GGT to PA ratio (GPR) has emerged as a potentially efficient indicator for prognostic prediction. Previous research has proposed the GPR as a novel marker for predicting the prognosis of HCC patients receiving locoregional ablative therapies [13]. However, evidence regarding its efficacy in predicting outcomes for patients undergoing hepatectomy is limited. Therefore, this study aimed to determine the predictive value of the GPR for prognosis in HCC patients and sought to construct nomograms tailored for clinical applications in prognostication.

## Materials and methods

### Data sources and population

HCC patients who underwent radical hepatectomy at Shengjing Hospital of China Medical University from December 2012 to January 2021 were retrospectively analyzed. The study had a minimum follow-up duration of 12 months and a median follow-up duration of 46 months. The inclusion criteria were as follows: (1) pathologically confirmed HCC; (2) treated by radical resection; and (3) complete clinical and follow-up data. The exclusion criteria for patients were as follows: (1) presence of pathological microvascular invasion (MVI); (2) concurrent other malignant tumors; (3) presence of metastases; and (4) Child–Pugh liver function grade C. According to these criteria, a total of 355 patients were eligible for inclusion in the study.

### Clinical data acquisition and research endpoints

The study collected the following clinical data: demographic data including age, gender, body mass index (BMI); tumor characteristics such as tumor size, tumor number; laboratory and imaging data including alpha-fetoprotein (AFP) levels, Child–Pugh grade, presence of hepatopathy, liver cirrhosis status, and portal hypertension; and surgical data including hepatic inflow occlusion and blood loss. Hepatopathy focused on whether patients were infected with hepatitis B (HBV) or hepatitis C (HCV). Liver cirrhosis status and portal hypertension were diagnosed based on radiological examination or pathological results. Additionally, the Barcelona Clinic Liver Cancer (BCLC) stage for each patient was evaluated and recorded. The endpoints of the study were progression-free survival (PFS) and overall survival (OS), defined as the time from the end of surgery to disease progression or death, respectively, or until the final follow-up time.

### Statistical analysis

All statistical analyses were conducted using R (version 4.4.0). Patients were randomly divided into a training cohort and a validation cohort at a ratio of 3:1. The training cohort was used for developing predictive models, while the validation cohort was employed for further verification. Continuous variables were assessed through t-tests and are presented as mean values with standard deviations. Categorical variables were analyzed via the chi-square test and were expressed as frequencies (percentages). The linearity of the GPR was assessed using restricted cubic spline (RCS) analysis, and the determination of the cut-off value was confirmed by X-tile, a software developed by Yale University for determining cut-off values for survival analysis [14]. Kaplan–Meier (KM) survival curves were generated to explore the associations between GPR and both PFS and OS, which were verified by the log-rank test. Univariate analysis identified potential risk factors, with variables having a significance level of *P* < 0.1 included in Cox multivariate stepwise analysis. Nomograms for PFS and OS were constructed based on the Cox model. Calibration curves and time-dependent receiver operating characteristic (ROC) curves were used to evaluate the prognostic predictive efficiency of the nomograms. Decision curve analysis (DCA) was conducted to assess the clinical application value of the models. Throughout the analysis, a significance threshold of *P* < 0.05 was considered statistically significant.

## Results

### Clinical characteristics of the populations

**Fig. 1:**
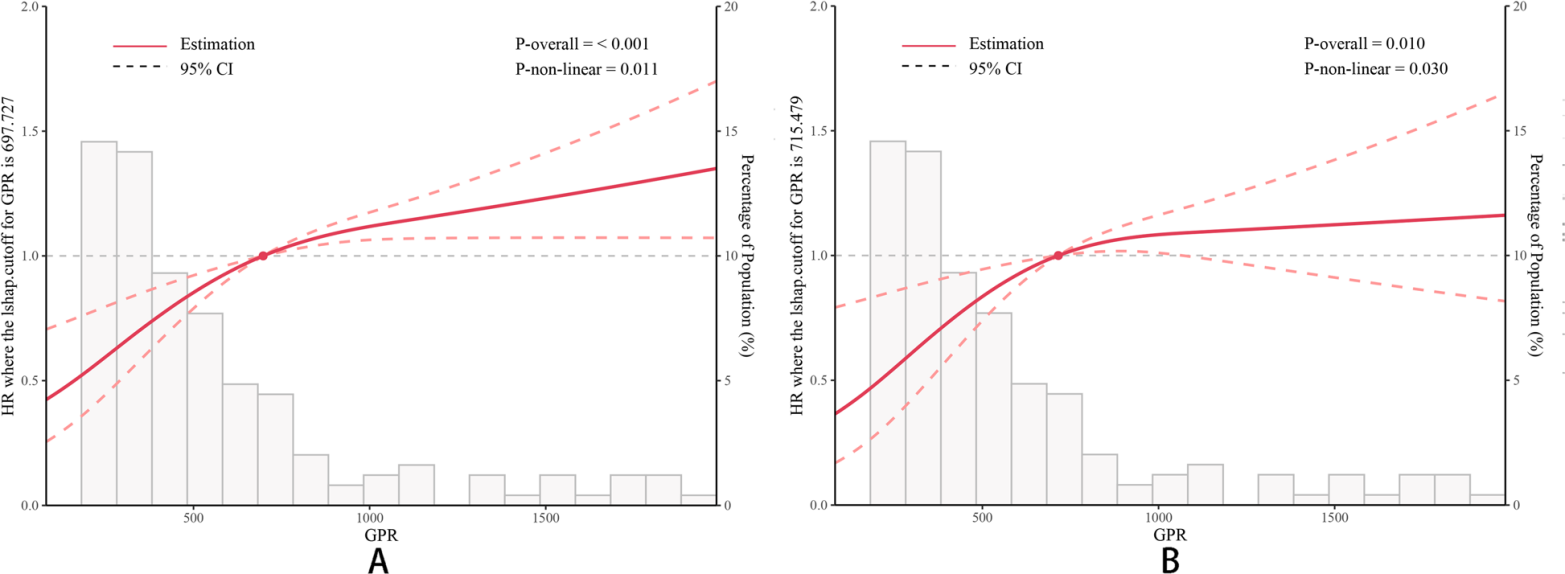
RCS of GPR for PFS and OS. **A** RCS for PFS; **B** RCS for OS

The 355 patients included in the study were randomly divided into a training cohort (*n* = 267) and a validation cohort (*n* = 88), with no significant differences observed between the two cohorts, as shown in Table 1. Therefore, the training cohort was deemed suitable for modeling purposes. The RCS analysis revealed a nonlinear relationship between GPR and both PFS (Fig. 1A) and OS (Fig. 1B). The graphical representation depicted an inverted L-shape, indicating a rapid escalation in the risk of relapse and death with the initial increase in GPR, followed by a slower increase or even a plateau after passing inflection points. Thus, it was imperative to categorize GPR as a binary variable for subsequent analysis. The X-tile software determined the cut-off value for GPR to be 333.33. Subsequently, both the training and validation cohorts were divided into GPR-low and GPR-high groups.

**Table 1:** Comparison of baseline characteristics between the training cohort and validation cohort

Variables	Total	Training cohort	validation cohort	*P*
	*n* = 355	*n* = 267	*n* = 88	
Age (years)	56.50 ± 9.86	56.70 ± 9.64	55.89 ± 10.52	0.466
Age category, n (%)				0.392
< 60 years	206 (58.03)	151 (56.55)	55 (62.50)	
≥ 60 years	149 (41.97)	116 (43.45)	33 (37.50)	
Gender, n (%)				0.216
Male	78 (21.97)	54 (20.22)	24 (27.27)	
Female	277 (78.03)	213 (79.78)	64 (72.73)	
BMI category, n (%)				0.547
≤ 24 kg/m^2^	202 (56.90)	149 (55.81)	53 (60.23)	
> 24 kg/m^2^	153 (43.10)	118 (44.19)	35 (39.77)	
Tumor size (cm)	4.98 ± 3.47	4.83 ± 3.23	5.42 ± 4.08	0.436
Tumor size category, n (%)				0.273
≤ 5 cm	229 (64.51)	177 (66.29)	52 (59.09)	
> 5 cm	126 (35.49)	90 (33.71)	36 (40.91)	
Tumor number, n (%)				0.364
Single	303 (85.35)	231 (86.52)	72 (81.82)	
Multiple	52 (14.65)	36 (13.48)	16 (18.18)	
AFP category				1
≤ 400 ng/mL	276 (77.75)	208 (77.90)	68 (77.27)	
> 400 ng/mL	79 (22.25)	59 (22.10)	20 (22.73)	
Child–Pugh grade, n (%)				1
A	341 (96.06)	256 (95.88)	85 (96.59)	
B	14 (3.94)	11 (4.12)	3 (3.41)	
HBV, n (%)				0.311
No	100 (28.17)	71 (26.59)	29 (32.95)	
Yes	255 (71.83)	196 (73.41)	59 (67.05)	
HCV, n (%)				1
No	334 (94.08)	251 (94.01)	83 (94.32)	
Yes	21 (5.92)	16 (5.99)	5 (5.68)	
Cirrhosis, n (%)				0.315
No	104 (29.30)	74 (27.72)	30 (34.09)	
Yes	251 (70.70)	193 (72.28)	58 (65.91)	
Portal hypertension, n (%)				0.784
No	280 (78.87)	212 (79.40)	68 (77.27)	
Yes	75 (21.13)	55 (20.60)	20 (22.73)	
Hepatic inflow occlusion, n (%)				0.638
No	163 (45.92)	125 (46.82)	38 (43.18)	
Yes	192 (54.08)	142 (53.18)	50 (56.82)	
Blood loss (mL)	286.96 ± 368.30	256.99 ± 267.4	377.56 ± 567.04	0.063
Blood loss category, n (%)				0.846
< 400 mL	242 (68.17)	184 (68.91)	59 (67.05)	
≥ 400 mL	113 (31.83)	83 (31.09)	29 (32.95)	
BCLC stage, n (%)				0.577
0	49 (13.80)	38 (14.23)	11 (12.50)	
A	275 (77.46)	208 (77.90)	67 (76.14)	
B	31 (8.73)	21 (7.87)	10 (11.36)	
GPR	531.16 ± 798.45	533.3 ± 838.6	524.69 ± 666.22	0.709

*Abbreviations*: *BMI* body mass index, *AFP* alpha-fetoprotein, *HBV* hepatic B virus, *HCV* hepatic C virus, *BCLC* Barcelona Clinic Liver Cancer, *GPR*: Gamma-glutamyl transpeptidase to prealbumin ratio

The baseline clinical characteristics of patients in the GPR-low group and the GPR-high group are summarized in Table 2. In the training cohort, significant differences were observed in the tumor size category, AFP category, Child–Pugh grade, portal hypertension, and blood loss category between the GPR-low and GPR-high groups. Similarly, in the validation cohort, significant differences were noted in the tumor size category and blood loss category between the GPR-low and GPR-high groups. The GPR-high group exhibited larger tumor size, worse Child–Pugh grade, a greater incidence of portal hypertension and larger amount of blood loss compared to the GPR-low group.

**Table 2:** Comparison of baseline characteristics between GPR-low and GPR-high groups in training and validation cohorts

	**Training cohort (*n* = 267)**			**Validation cohort (*n* = 88)**		
**Variables**	**GPR-low**	**GPR-high**	***P***	**GPR-low**	**GPR-high**	***P***
	*n* = 150	*n* = 117		*n* = 52	*n* = 36	
Age category, n (%)			0.507			0.654
<60 years	88 (58.67)	63 (53.85)		31 (59.62)	24 (66.67)	
≥60 years	62 (41.33)	54 (46.15)		21 (40.38)	12 (33.33)	
Gender, n (%)			0.201			0.106
Male	35 (23.33)	19 (16.24)		18 (34.62)	6 (16.67)	
Female	115 (76.67)	98 (83.76)		34 (65.38)	30 (83.33)	
BMI category, n (%)			0.959			0.064
≤24 kg/m^2^	83 (55.33)	66 (56.41)		36 (69.23)	17 (47.22)	
>24 kg/m^2^	67 (44.67)	51 (43.59)		16 (30.77)	19 (52.78)	
Tumor size category, n (%)			**0.002**			**0.011**
≤ 5 cm	112 (74.67)	65 (55.56)		37 (71.15)	15 (41.67)	
> 5 cm	38 (25.33)	52 (44.44)		15 (28.85)	21 (58.33)	
Tumor number, n (%)			0.237			0.980
Single	126 (84.00)	105 (89.74)		42 (80.77)	30 (83.33)	
Multiple	24 (16.00)	12 (10.26)		10 (19.23)	6 (16.67)	
AFP category, n (%)			**0.048**			0.086
≤400 ng/mL	124 (82.67)	84 (71.79)		44 (84.62)	24 (66.67)	
>400 ng/mL	26 (17.33)	33 (28.21)		8 (15.38)	12 (33.33)	
Child-Pugh grade, n (%)			**0.004**			0.128
A	149 (99.33)	107 (91.45)		52 (100.00)	33 (91.67)	
B	1 (0.67)	10 (8.55)		0 (0.00)	3 (8.33)	
HBV, n (%)			0.344			0.529
No	36 (24.00)	35 (29.91)		19 (36.54)	10 (27.78)	
Yes	114 (76.00)	82 (70.09)		33 (63.46)	26 (72.22)	
HCV, n (%)			1			0.609
No	141 (94.00)	110 (94.02)		48 (92.31)	35 (97.22)	
Yes	9 (6.00)	7 (5.98)		4 (7.69)	1 (2.78)	
Cirrhosis, n (%)			1			0.417
No	42 (28.00)	32 (27.35)		20 (38.46)	10 (27.78)	
Yes	108 (72.00)	85 (72.65)		32 (61.54)	26 (72.22)	
Portal hypertension, n (%)			**0.004**			1
No	129 (86.00)	83 (70.94)		40 (76.92)	28 (77.78)	
Yes	21 (14.00)	34 (29.06)		12 (23.08)	8 (22.22)	
Hepatic inflow occlusion, n (%)			0.946			1
No	71 (47.33)	54 (46.15)		22 (42.31)	16 (44.44)	
Yes	79 (52.67)	63 (53.85)		30 (57.69)	20 (55.56)	
Blood loss category, n (%)			**0.003**			**<0.001**
<400 mL	115 (76.67)	69 (58.97)		44 (84.62)	15 (41.67)	
≥400 mL	35 (23.33)	48 (41.03)		8 (15.38)	21 (58.33)	
BCLC stage, n (%)			0.249			0.241
0	26 (17.33)	12 (10.26)		9 (17.31)	2 (5.56)	
A	112 (74.67)	96 (82.05)		38 (73.08)	29 (80.56)	
B	12 (8.00)	9 (7.69)		5 (9.62)	5 (13.89)	

*Abbreviations*: *GPR* Gamma-glutamyl transpeptidase to prealbumin ratio, *BMI* body mass index, *AFP* alpha-fetoprotein, *HBV* hepatic B virus, *HCV* hepatic C virus, *BCLC* Barcelona Clinic Liver Cancer

### KM survival curves of GPR for OS and PFS

**Fig. 2:**
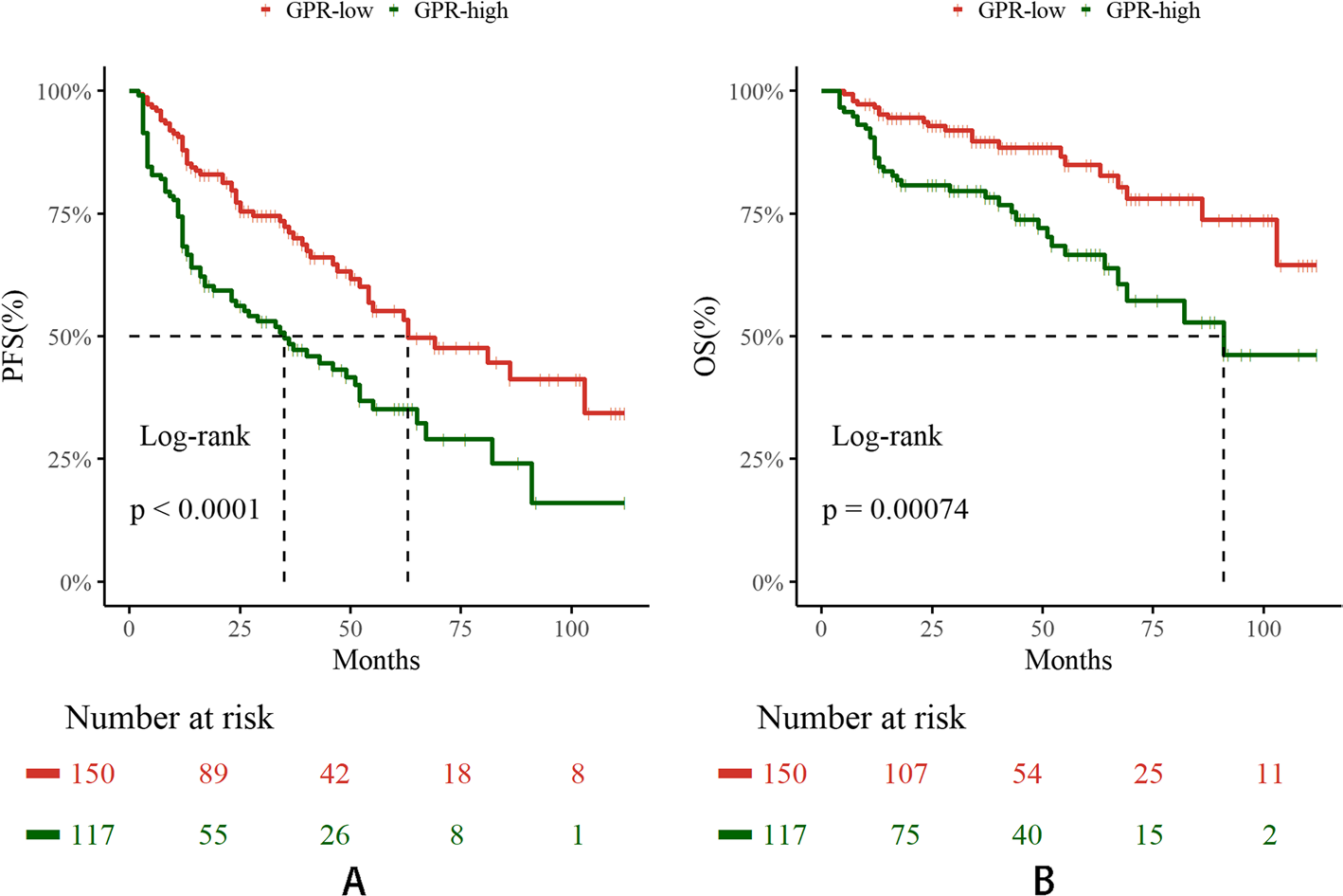
KM curves of GPR for OS and PFS. **A** PFS; **B** OS

The KM curves are shown in Fig. 2. Notably, the GPR-high group exhibited significantly poorer PFS and OS outcomes compared to the GPR-low group, with statistical significance observed (both *P* < 0.001). The median PFS durations for the GPR-low and GPR-high groups were 63 and 35 months, respectively. Additionally, it is noteworthy that only the GPR-high group reached the median OS of 91 months, whereas the GPR-low group did not reach the median OS.

### Univariate and multivariate analyses

**Fig. 3:**
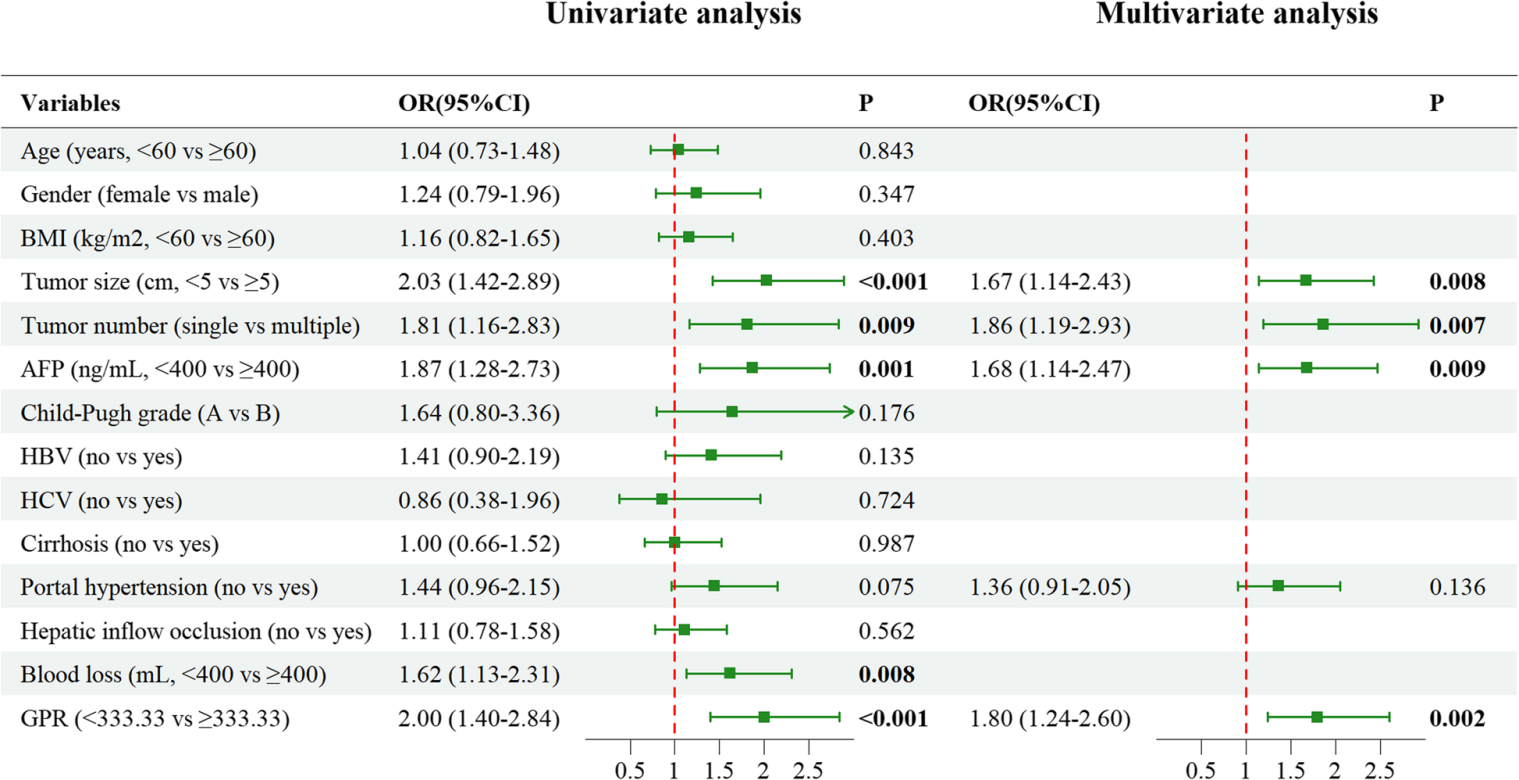
Univariate and multivariate analysis of PFS

**Fig. 4:**
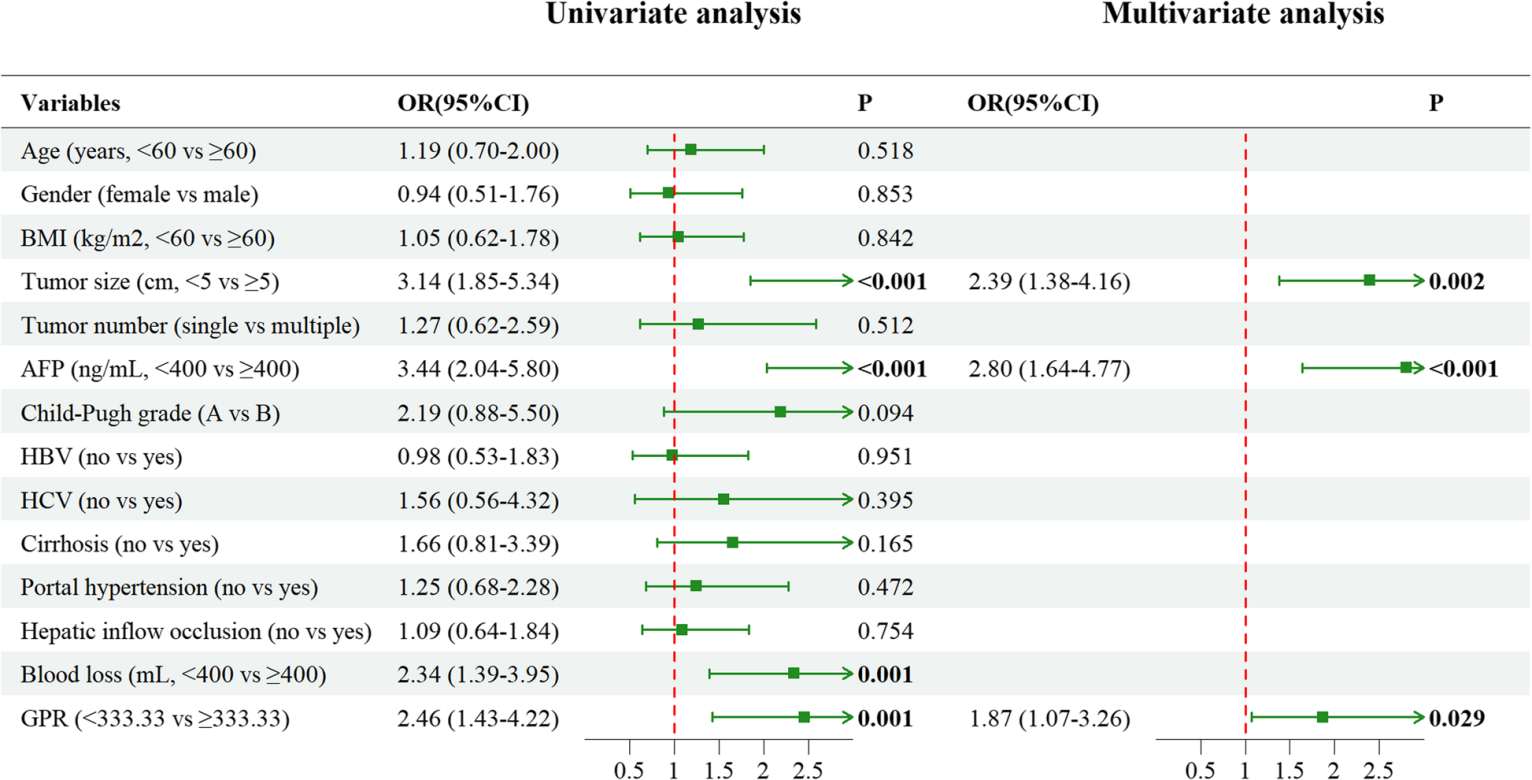
Univariate and multivariate analysis of OS

The results of the univariate analysis and multivariate analysis for both PFS and OS are depicted in Fig. 3 and Fig. 4, respectively. Variables with a significance level of *P* < 0.1 in the univariate analysis were included in the multivariate analysis. To enhance visualization, all continuous variables were converted into categorical variables in both univariate and multivariate analyses. Utilizing the Akaike information criterion (AIC) principle, we employed Cox stepwise regression analysis for variable selection. Ultimately, tumor size (OR = 1.67, 95% CI: 1.14–2.43, *P* = 0.008), tumor number (OR = 1.86, 95% CI: 1.19–2.93, *P* = 0.007), AFP (OR = 1.68, 95% CI: 1.14–2.47, *P* = 0.009), portal hypertension (OR = 1.36, 95% CI: 0.91–2.05, *P* = 0.136), and GPR (OR = 1.80, 95% CI: 1.24–2.60, *P* = 0.002) were included in the predictive model for PFS. In terms of OS, tumor size (OR = 2.39, 95% CI: 1.38–4.16, *P* = 0.002), AFP (OR = 2.80, 95% CI: 1.64–4.77, *P* < 0.001), and GPR (OR = 1.87, 95% CI: 1.07–3.26, *P* = 0.029) were identified as significant predictors.

### Predictive models and evaluation

**Fig. 5:**
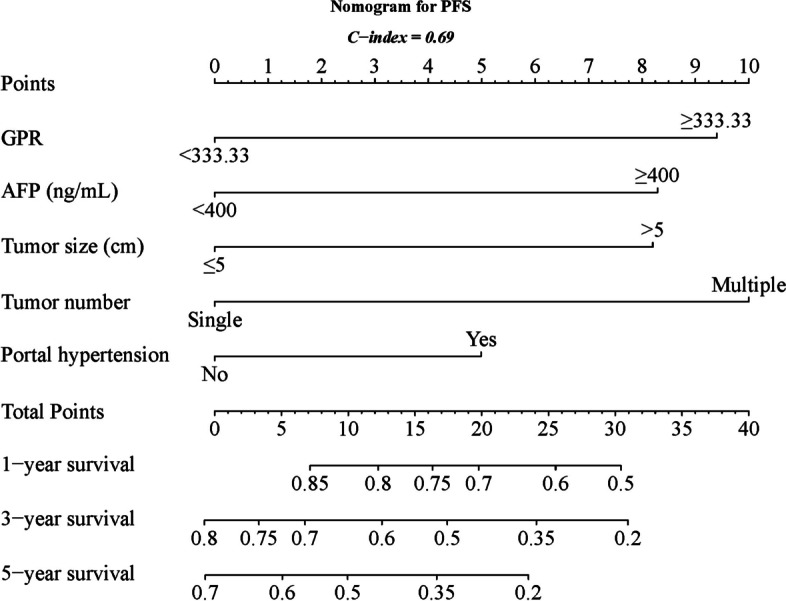
Nomogram for PFS

**Fig. 6:**
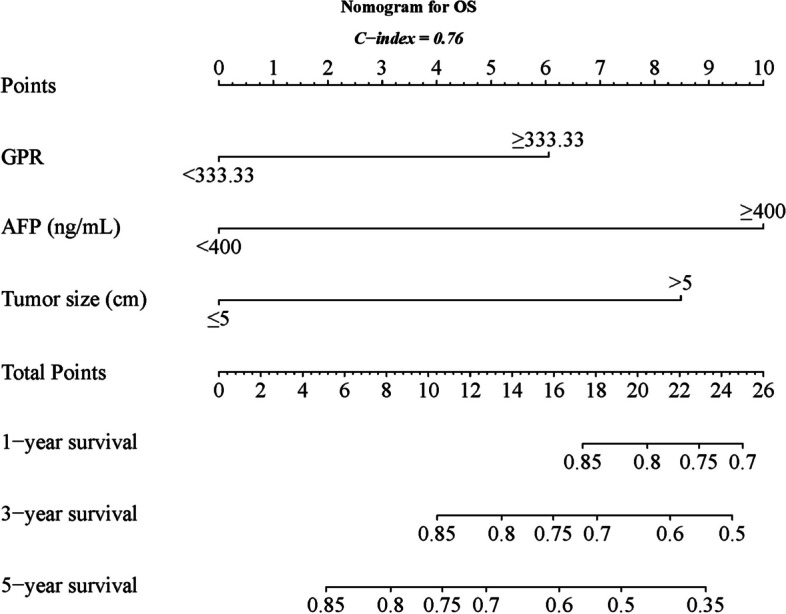
Nomogram for OS

Based on insights from the multivariate analysis, nomograms were developed and visually presented in Fig. 5 for PFS and Fig. 6 for OS. The C-index, a metric of predictive accuracy, yielded values of 0.69 for PFS and 0.76 for OS, confirming the reliability of the nomograms.

**Fig. 7:**
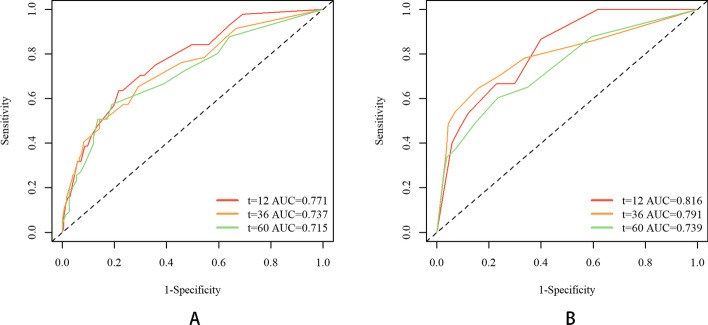
Time-dependent ROC curves in the training cohort. **A** PFS; **B** OS

**Fig. 8:**
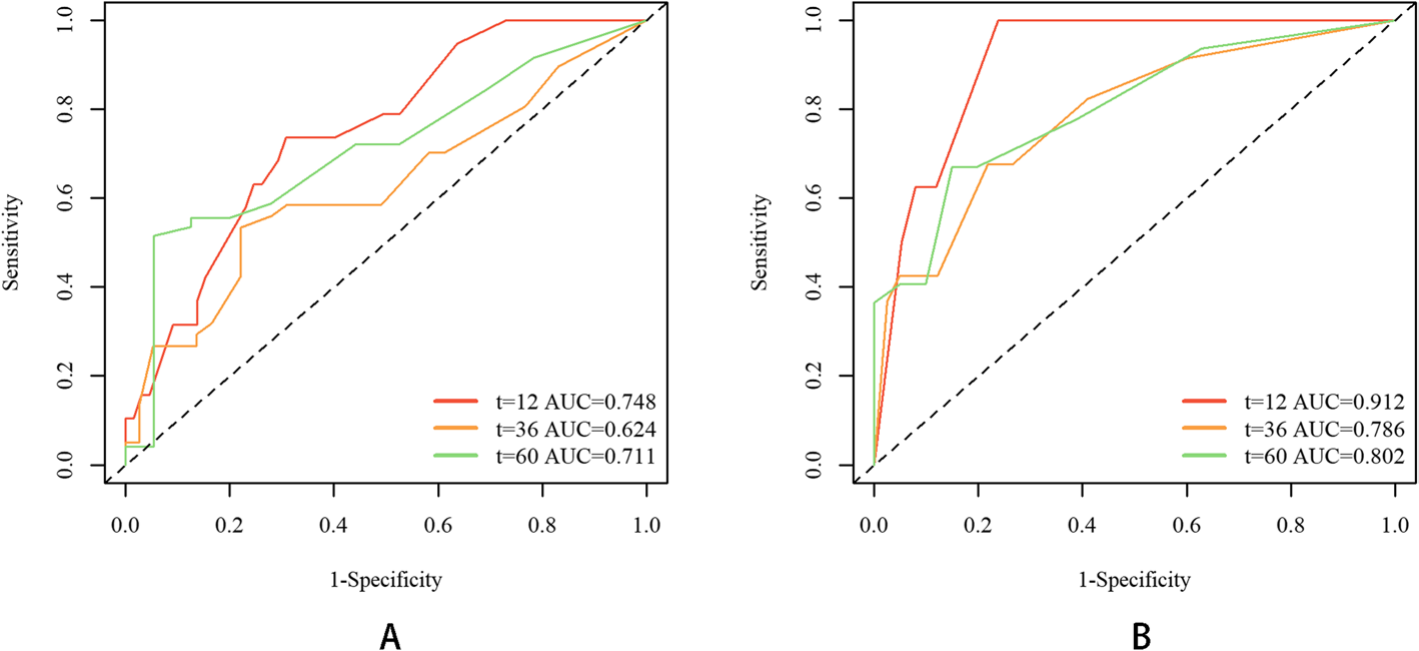
Time-dependent ROC curves in the validation cohort. **A** PFS; **B** OS

Time-dependent ROC curves revealed the robust predictive ability of the two models. As displayed in Fig. 7, the 1-, 3-, and 5-year areas under the curves (AUCs) of the time-dependent ROC curves were 0.771, 0.737, and 0.715 for PFS and 0.816, 0.791, and 0.739 for OS, respectively, in the training cohort. Similarly, for the validation cohort, the 1-, 3-, and 5-year AUCs of the time ROC curve were 0.748, 0.624, and 0.711 for PFS and 0.912, 0.786, and 0.802 for OS, respectively, as shown in Fig. 8.

**Fig. 9:**
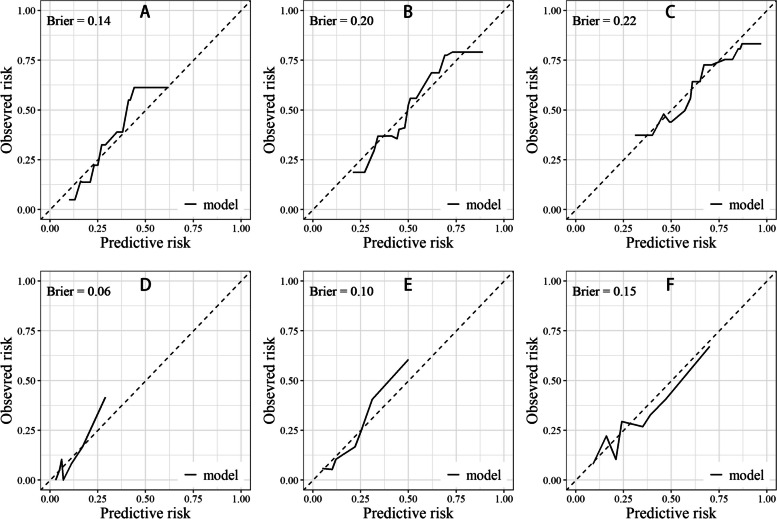
Calibration curves in the training cohort. **A**, **B** and (**C**): 1-, 3-, and 5-years calibration curve for PFS; **D**, **E** and **F**: 1-, 3-, and 5-years calibration curve for OS

**Fig. 10:**
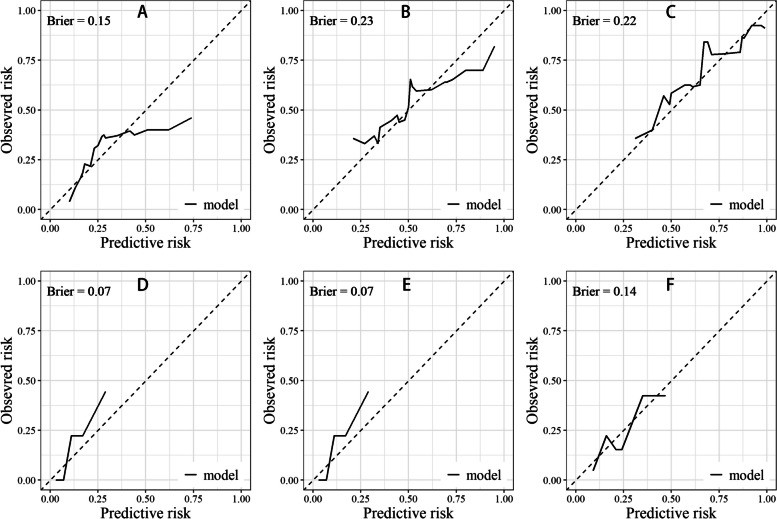
Calibration curves in the validation cohort. **A**, **B** and **C**: 1-, 3-, and 5-years calibration curve for PFS; **D**, **E** and **F**: 1-, 3-, and 5-years calibration curve for OS

The calibration curves provided additional evidence of the models’ accuracy, revealing a close alignment between the predicted and observed outcomes. As depicted in Fig. 9 and Fig. 10, all of the Brier scores for each calibration curve were less than 0.25, indicating a high level of prognostic prediction efficiency.

**Fig. 11:**
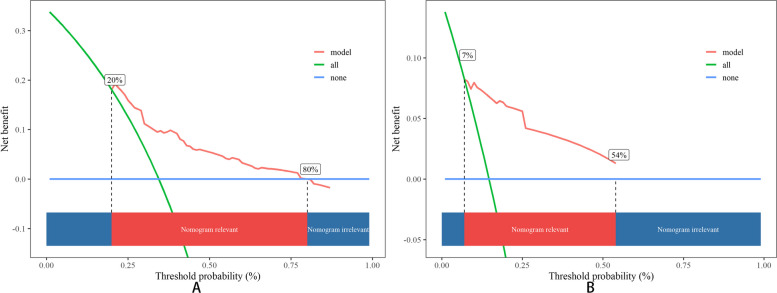
DCA in the training cohort. **A** PFS; **B** OS

**Fig. 12:**
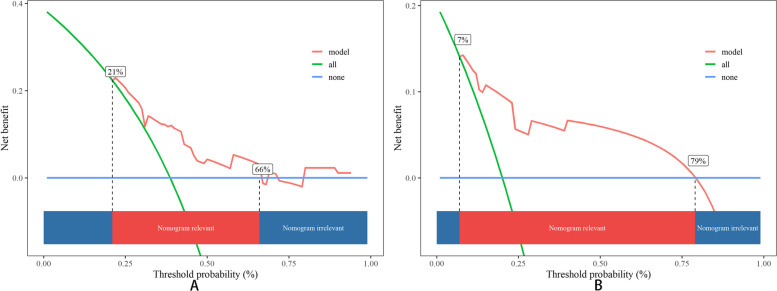
DCA in the validation cohort. **A** PFS; **B** OS

In-depth assessment through DCA, as illustrated in Fig. 11 and Fig. 12, revealed that both the PFS and OS nomograms delivered substantial benefits compared to the simplistic strategies such as "treat-all" and "treat-none",a relatively good threshold.

## Discussion

In our study, we identified the GPR as an independent risk factor for both PFS and OS in HCC patients without MVI who underwent radical resection. Our results revealed a nonlinear correlation between GPR and prognosis, characterized by an initial rapid impact followed by a gradual trend and eventually a near-plateau period. The predictive nomograms incorporating GPR exhibited significant efficiency, highlighting their potential applicability in clinical practice. Previous research has also demonstrated that a higher GPR is associated with poor prognosis in HCC patients receiving ablation, thereby corroborating our findings [13]. Consequently, given the high postoperative relapse rate in HCC, these nomograms developed in this study provide valuable assistance to clinicians in designing personalized postoperative monitoring plans and informed treatment strategies.

Our study also revealed that compared to the GPR-low group, the GPR-high group exhibited worse Child-Pugh grade and a greater incidence of portal hypertension. This association can be attributed to the fact that abnormal liver function and cholestasis often lead to elevated levels of GGT, a component of the GPR. Elevated GGT levels have been implicated in tumor formation and progression through various mechanisms [15]. Consequently, previous studies have recommended utilizing the GGT to platelet ratio as a prognostic marker for HCC patients [16, 17]. Additionally, individuals with malignant tumors often experience compromised nutritional status due to heightened metabolism and chronic inflammation [18], leading to decreased PA levels. Further exploration is needed to fully understand the deep relationship between GGT levels and the prognosis of HCC patients. The GPR can not only reflect liver function damage and the nutritional status of HCC patients but also indicate the degree of tumor proliferation. This multifaceted perspective may elucidate the rationale behind the prognostic predictive ability of the GPR in HCC patients.

In our study, we observed significant associations between both nomograms and tumor characteristics, as well as AFP levels. AFP, recognized as the most widely used tumor biomarker for HCC, plays a crucial role in the progression of HCC [19]. Additionally, tumor size and number are well-established prognostic factors, with larger tumor size and increased tumor number indicating poorer outcomes [20, 21]. Furthermore, our study revealed that the GPR-high group exhibited larger tumor size compared to the GPR-low group. Moreover, tumor size and number significantly affected PFS, and tumor size affected OS. These findings were largely consistent with previous studies, thus bolstering and broadening our comprehension. This coherence fortifies the reliability and applicability of our results, underscoring the reproducibility of predictive nomograms reliant on GPR.

Our study has several strengths. To our knowledge, this is the first study to highlight GPR as an independent predictive factor for the prognosis of HCC patients without MVI undergoing radical resection. The construction of predictive models based on Cox analyses, coupled with the development of nomograms, enhances the precision of clinical prognosis assessments. The demonstrated C-index values underscore the efficacy of the nomograms in terms of prediction accuracy, while sensitivity analysis further validated the models, confirming their relatively robust predictive efficiency.

However, there are still some limitations to acknowledge. Firstly, this study was conducted as a single-center retrospective analysis, which may introduce sample bias and limit the generalizability of our findings. Secondly, although the sample size was carefully considered and internally validated, additional external validation through multicenter large-sample prospective analyses is necessary to confirm the robustness of our results across diverse patient populations. Lastly, the inclusion of non-statistically significant variables in both PFS nomogram requires careful consideration. Continued exploration and statistical analysis are warranted to determine the suitability of the variable for refining predictive models and improving prognostic accuracy.

## Conclusion

In conclusion, our study highlights the potential of the GPR as an independent risk factor for both PFS and OS in HCC patients without MVI. The nomograms developed in this study provide clinicians with valuable tools to aid in the formulation of personalized reexamination strategies and treatment protocols.

## Declaration of Generative AI and AI-assisted technologies in the writing process

During the preparation of this work, the authors used ChatGPT to polish this article. After using this tool, the authors reviewed and edited the content as needed and took full responsibility for the publication's content.

## Data Availability

The datasets utilized in the present study can be obtained from the corresponding author upon reasonable request.

## Abbreviations

HCC: Hepatocellular carcinoma
GGT: Gamma-glutamyl transpeptidase
PA: Prealbumin
GPR: Gamma-glutamyl transpeptidase to prealbumin ratio
MVI: Microvascular invasion
KM: Kaplan–Meier (KM) survival curves
ROC: Receiver operating characteristic
BMI: Body mass index
AFP: Alpha-fetoprotein
HBV: Hepatitis B
HCV: Hepatitis C
BCLC: Barcelona Clinic Liver Cancer
PFS: Progress-free survival
OS: Overall survival
RCS: Restricted cubic spline
DCA: Decision curve analysis
AIC: Akaike information criterion
AUC: Areas under curve
